# Ultra-High-Resolution Mass Spectrometry for Identification of Closely Related Dermatophytes with Different Clinical Predilections

**DOI:** 10.1128/JCM.00102-18

**Published:** 2018-06-25

**Authors:** Karolina Dukik, Joanna Freeke, Azadeh Jamalian, Bert Gerrits van den Ende, Ping Yip, James L. Stephenson, G. Sybren de Hoog, J. Benjamin Stielow

**Affiliations:** a Westerdijk Fungal Biodiversity Institute, Utrecht, The Netherlands; b Institute for Biodiversity and Ecosystem Dynamics, University of Amsterdam, Amsterdam, The Netherlands; c Thermo Fisher Scientific, Landsmeer, The Netherlands; d Thermo Fisher Scientific, Cambridge, Massachusetts, USA

**Keywords:** clinical mycology, dermatophytes, identification, LC-MS/MS, Trichophyton

## Abstract

In the present study, an innovative top-down liquid chromatography-tandem mass spectrometry (LC-MS/MS) method for the identification of clinically relevant fungi is tested using a model set of dermatophyte strains. The methodology characterizes intact proteins derived from Trichophyton species, which are used as parameters of differentiation. To test its resolving power compared to that of traditional Sanger sequencing and matrix-assisted laser desorption ionization–time of flight mass spectrometry (MALDI-TOF), 24 strains of closely related dermatophytes, Trichophyton rubrum, T. violaceum, T. tonsurans, T. equinum, and T. interdigitale, were subjected to this new approach. Using MS/MS and different deconvolution algorithms, we identified hundreds of individual proteins, with a subpopulation of these used as strain- or species-specific markers. Three species, i.e., T. rubrum, T. violaceum, and T. interdigitale, were identified correctly down to the species level. Moreover, all isolates associated with these three species were identified correctly down to the strain level. In the T. tonsurans-equinum complex, eight out of 12 strains showed nearly identical proteomes, indicating an unresolved taxonomic conflict already apparent from previous phylogenetic data. In this case, it was determined with high probability that only a single species can be present. Our study successfully demonstrates applicability of the mass spectrometric approach to identify clinically relevant filamentous fungi. Here, we present the first proof-of-principle study employing the mentioned technology to differentiate microbial pathogens. The ability to differentiate fungi at the strain level sets the stage to improve patient outcomes, such as early detection of strains that carry resistance to antifungals.

## INTRODUCTION

Dermatophytes are fungi that are able to invade keratinized tissues, causing infections of the skin, hair, and nails (1). Almost every human contracts at least one such infection in their lifetime. Due to this high incidence, over 500 million dollars are spent annually on antimycotic treatment against dermatophytes (2). The prevalent species encountered in dermatology are classified in three genera: Trichophyton, Microsporum, and Epidermophyton. Trichophyton in a modern sense comprises the anthropophilic species, along with species infecting domesticated animals (3).

In the routine clinical laboratory, the presentation of clinical symptoms, colony morphology, microscopic features, physiology, or, alternatively, nucleic acid sequencing of the internal transcribed spacer (ITS) domain are commonly applied to dermatophyte identification. Following its successful adoption by many clinical laboratories, matrix-assisted laser desorption ionization–time of flight mass spectrometry (MALDI-TOF) mass spectrometry has been applied to a broad range of species (4–9) or species groups of dermatophytes (10, 11). Even with these recent advances in identification and characterization using MALDI-TOF, accuracy rates remain at the 50 to 60% range with very high no-call rates (12), which is partly due to inadequate taxonomy of dermatophytes at the DNA level (3). In other fungal groups, identification of clinically relevant filamentous fungi has been shown to be possible with the addition of custom acquired data supplementing IVD databases (6, 7).

As an alternative strategy to the MALDI-TOF fingerprint approach, proteome-based strategies involve identification of proteins derived from microbial extracts. Two fundamentally different mass spectrometric strategies are available for protein identification: bottom-up and top-down. In bottom-up proteomics, purified proteins or complex protein mixtures are subjected to proteolytic cleavage prior to MS analysis. In top-down proteomics, intact protein ions or large protein fragments are injected directly into the mass spectrometer, where they are further fragmented. The main advantage of top-down analysis is the ability to reveal intact protein masses, structural amino acid sequence variants, and (combinations of) posttranslational modifications.

In the present study, we utilize liquid chromatography-tandem mass spectrometry (LC-MS/MS) to separate proteins from dermatophyte extracts and analyze them sequentially in an Orbitrap tandem mass spectrometer. Amino acid sequence information obtained from tandem mass spectrometry is used to identify the observed proteins, which in turn leads to the correct classification of clinically relevant dermatophytes. This MS/MS process, termed collision-induced dissociation (CID), imparts excess energy to the intact protein ions, resulting in smaller-mass amino acid sequence-specific protein fragments which are used to directly identify any given protein undergoing this process. Several thousand highly informative MS/MS spectra from the fragmented proteins or peptides are obtainable this way in a single run. The key difference of this approach compared to fingerprinting/pattern recognition by MALDI-TOF is the accurate assignment of intact protein and fragment masses that allows for statistically relevant high-confidence protein identification (13). In turn, these identified proteins, either singly or in combination, can be used as diagnostic markers of clinically relevant microorganisms.

**FIG 1:**
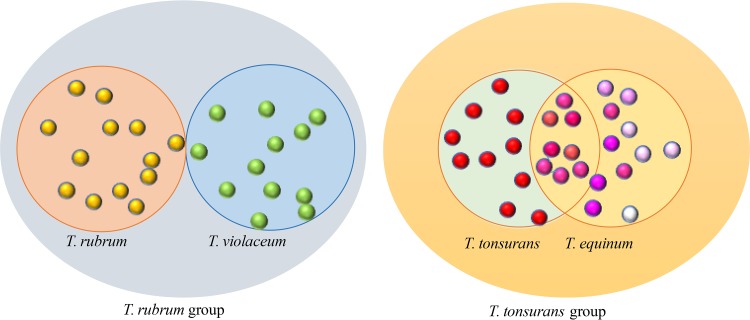
Two species groups, T. rubrum with T. violaceum and T. tonsurans with T. equinum. The members of the T. rubrum group are considered to be different, whereas the separation of the other pair is doubtful, possibly involving only a single species.

The goal of the present study was to provide a proof-of-principle experiment employing Orbitrap LC-MS/MS for discrimination of filamentous fungi and to establish a proteomic approach for detailed characterization of strain diversity of the investigated taxa. As a model, two closely related but different pairs of species were compared. The members of one species pair are known to belong to unambiguously different species, whereas the separation of the other pair or lineages is doubtful, possibly comprising only a single species (Fig. 1). The former set concerns the Trichophyton rubrum group, comprising two species: T. rubrum, with a global prevalence and mainly causing tinea corporis and tinea pedis, and T. violaceum, which mostly causes tinea capitis and is endemic to northern Africa and the Middle East. Trichophyton soudanense belongs to the latter group but is generally judged a synonym of T. violaceum (14). This set is compared to the Trichophyton tonsurans complex, which comprises two lineages that are often regarded as synonymous (15): T. tonsurans and T. equinum. The former is an anthropophilic entity causing tinea capitis in humans, while its zoophilic counterpart, T. equinum, causes ringworm in horses but is also found in humans (3).

Our study successfully demonstrates a solution to a long-existing technical challenge, i.e., the possibility of employing liquid chromatography coupled with ultra-high-resolution Orbitrap mass spectrometry for microbial species identification. Massive quantities of fully resolved individual microbial proteins render Orbitrap mass spectrometry several orders of magnitude higher in sensitivity and specificity than currently existing proteomic technologies. Subsequently this will set the stage to improve patient care, significantly enabling microbial identification down to the strain level.

## MATERIALS AND METHODS

### Strains and growth conditions.

Strains studied were acquired from the reference collection of Centraalbureau voor Schimmelcultures at the Westerdijk Fungal Biodiversity Institute (Table 1). Strains were part of a taxonomic study applying multilocus sequencing (3) and included (neo)type strains of synonymized species Trichophyton raubitschekii, T. rubrum var. nigricans, T. fischeri, T. soudanense, and T. violaceum in the T. rubrum group and Trichophyton areolatum, T. floriforme, T. equinum, and T. equinum var. autotrophicum in the T. tonsurans group. Two strains of T. interdigitale were included as closest relatives of T. tonsurans, serving as a marker of nonidentity. Nine out of 12 strains in this group had variously been classified as either T. tonsurans or T. equinum (Table 1). Lyophilized or cryopreserved material was activated on Sabouraud's glucose agar plates (SGA; Oxoid, Thermo Scientific) and incubated at 24°C for 3 weeks due to slow growth of T. violaceum.

**TABLE 1:** Trichophyton strains analyzed in this study

Trichophyton strain^*a*^	Taxonomy change^*b*^	Source	Clinical picture	Country	GenBank accession no.
T. rubrum CBS 115314		Human	Onychomycosis	Greece	KT155714
T. rubrum CBS 100084 (T)	T. raubitschekii	Human	Skin	Canada	KT155667
T. rubrum CBS 1000238	T. rubrum var. nigicans	Human			KT155669
T. rubrum CBS 288.86	T. fischeri	Contaminant		Canada	AJ270793
T. rubrum CBS 202.88	T. raubitschekii	Human	Tinea pedis	Canada	AJ270804
T. rubrum CBS 118892		Human	Onychomycosis	Germany	KT155731
T. violaceum CBS 120320		Human	Tinea capitis	Switzerland	KT155740
T. violaceum CBS 120316		Human	Tinea capitis	Switzerland	KT155737
T. violaceum CBS 201.88	T. soudanense	Human	Tinea faciei	Canada	KT310173
T. violaceum CBS 452.61	T. soudanense	Human	Endothirx variants and tinea capitis	Zaire	AJ270809
T. tonsurans CBS 285.30 (T)	T. areolatum	Human	Endrothrix	Argentina	KT155645
T. tonsurans CBS 318.31 (T)	T. floriforme	Human			KT310170
T. tonsurans CBS 856.71	T. equinum var. equinum	Horse	Hair	The Netherlands	KT310172
T. tonsurans CBS 127.97	T. equinum, T equinum var. equinum	Human	Onychomycosis and tinea manuum	Finland	KT310169
T. tonsurans CBS 109033	T. equinum	Horse	Skin	Canada	KT155681
T. tonsurans CBS 112186		Human		England	KT155688
T. equinum CBS 270.66 (NT)	T. equinum var. equinum, T. tonsurans	Horse		USA	KT155643
T. equinum CBS 634.82	T. equinum var. autotrophicum, T. tonsurans	Horse	Tinea	New Zealand	KT310171
T. equinum CBS 100080 (T)	T. equinum var. autotrophicum, T. tonsurans	Horse		New Zealand	KT155665
T. equinum CBS 112188	T. tonsurans	Horse		England	EF043277
T. equinum CBS 112193	T. tonsurans	Horse		England	KT155693
T. equinum CBS 112198	T. tonsurans	Human		England	EF043275
T. interdigitale CBS 119447		Human	Tinea capitis	Gabon	KT155733
T. interdigitale CBS 120318		Human	Tinea capitis	Switzerland	KT155738

a T, type; NT, neotype.

b Data for taxonomy changes are all name changes recorded in the CBS database (with previous nomenclatural changes for a particular strain).

### DNA extraction, PCR, and sequencing.

Genomic DNA was extracted using Illumina's MasterPure DNA purification kit (Illumina) according to the manufacturer's protocol. Ribosomal DNA (rDNA) ITS was sequenced using ITS5 and ITS4 primers under standard conditions (16). PCR products were purified with FastAP thermo-sensitive alkaline phosphatase and shrimp alkaline phosphatase (Fermentas, Thermo Scientific). Sequencing reactions were done in 10-μl volumes using Thermo Scientific BigDye Terminator v.3.1 on a 3730XL instrument (Thermo Scientific). Sequences were deposited at NCBI GenBank (Table 1). Obtained sequences were manually edited, and consensus sequences were aligned with MAFFT v. 6.850b with default settings (17). Identification was performed by querying sequences against NCBI GenBank and the Westerdijk Institute website (www.westerdijkinstitute.nl).

### Protein extraction and purification.

Protein extractions were performed on three biological replicates per strain. Briefly, approximately 5 mg biomass was harvested with a scalpel from a culture plate and transferred to a microvial (Eppendorf) with lysis buffer containing formic acid and acetonitrile (proprietary ratios). Cells were disrupted and then centrifuged for 1 min at 14,000 rpm. The supernatant was then transferred to a new vial. Extracts were diluted to 10% acetonitrile and desalted using Lab_in_a_Plate plates (Glygen Corp., USA). Equilibration, loading, and washing steps were done according to the manufacturer's protocol, with minor changes. Samples were eluted in 40% acetonitrile with 0.1% formic acid.

### LC-MS/MS analysis, data processing, and identification.

Chromatographic separation was done by injecting 8 μl of the protein extracts on a Thermo Scientific EASY-Spray Accucore C_4_ column (15 cm, 75-μm inner diameter, 2.6-μm particles, and 150-Å pore size). Protein separation was achieved with a 1-h gradient starting with buffer A (0% acetonitrile, 0.1% formic acid) to 60% buffer B (60% acetonitrile and 0.1% formic acid) in 50 min at a column temperature of 60°C and a flow rate of 200 μl/min. The LC system was coupled with a Thermo Scientific Q Exactive Plus hybrid quadrupole mass spectrometer. Mass analysis was done with the top-down method of 5 microscans, a scan range of 350 to 2,000 Da, and loop counts for data-dependent (dd) analysis being 15.

**FIG 2:**
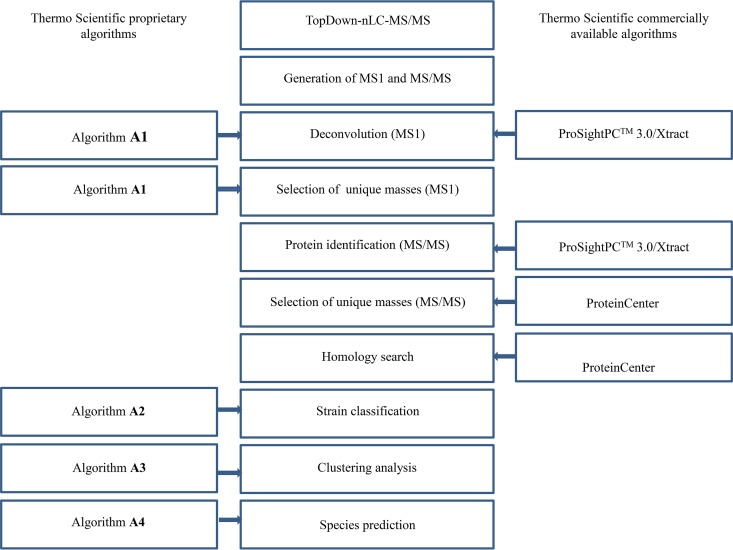
Overview of all Thermo Scientific algorithms employed in stepwise analysis of LC-MS/MS data. The center column indicates workflow from start (top) to end (bottom), with branching elements left/right indicating, e.g., the algorithm employed for data analysis (e.g., ProSightPC).

Algorithms used for further analysis of the acquired data are given in Fig. 2. Deconvolution of the mass spectra was performed via two algorithms. The first employed Thermo Fisher Scientific proprietary software (algorithm A1) to deconvolute raw spectra in *m/z* space into monoisotopic protein masses. An alternative approach was conducted via Thermo Scientific ProSightPC 3.0 for deconvolution of intact protein mass spectra and analysis of MS/MS fragment spectra (Thermo Scientific Xtract build-in). Subsequently, MS/MS fragment spectra were queried against a custom database obtained from UniProt (http://www.uniprot.org/) using ProSightPC 3.0 and containing amino acid sequences of the genera Trichophyton, Microsporum, Epidermophyton, and Arthroderma (883,412 predicted proteoforms in total). The queries were performed as absolute mass search (considering disulfide linkages and Δ*m* applied) in a specified intact mass window of 1,000 Da; selected parameters were defined as 15-ppm fragment mass tolerance, with acetylation and posttranslational modifications (PTM) applied as criteria and a cutoff expectation value (E value) of <0.0001. Identified protein sequences with a confidence (E value) score higher than 1.0 × 10^−4^ were further analyzed in the versatile custom sequence database and analysis software ProteinCenter (Thermo Fisher Scientific). Using ProteinCenter, the identified proteins were subjected to homology search using an 80% similarity cutoff in an attempt to find identical or similar sequences in other dermatophytes. Subsequent searches were constrained to 100% homology level to clear redundancies.

In addition to inferring species affiliations with identified protein sequences from MS/MS fragment spectra, we ran two additional analyses to classify the strains and to predict the species where the analyzed strains belong. An unreported Thermo Fisher proprietary classification algorithm (algorithm A2) inferred the strain classification analysis. The prediction was repeated four times in order to establish variance between replicates. The resulting classification accuracy has values from 0 to 1, with 1 being all three replicates of a strain that were correctly identified in all four predictions.

Species prediction was performed with algorithm A4. The same data were used to establish statistical independence over the current taxonomic species affiliation but constrained to a chosen reference strain to guide the species prediction. The analysis was conducted twice with different reference strains, each analysis with three iterations for the individual taxa. Two analyses were conducted with one or two reference strains. In the first analysis, one or two (neo)type strains or randomly chosen strains were used as reference strains to infer accuracy. Prior to the second analysis, we performed clustering of the 500 most consistently measured monoisotopic masses using a Thermo Scientific proprietary clustering algorithm, A3 (data not shown). Clustering of strains with these monoisotopic masses revealed which strains have the highest number of shared masses. Based on this criterion, one or two of these strains were chosen as references. The latter step was required to avoid atypical selections.

### Accession number(s).

Sequences determined in this work were deposited at NCBI GenBank and are listed in Table 1.

## RESULTS

### DNA-based identification.

All strains had been identified prior to protein analysis using rDNA ITS as a barcode. Nucleotide sequence differences were established by separately aligning the two complexes. In the T. rubrum complex, T. rubrum strains differed from T. violaceum (including T. soudanense) with 4 single-nucleotide polymorphisms (SNPs) at positions 167 (ITS1), 525, 543, and 544 (ITS2). Differences in the number of AT repeats at the end of ITS2 reported in the literature were not found to differ consistently in this alignment and therefore were not taken into account. The six ITS sequences of T. rubrum were identical. In T. violaceum, CBS 452.61, denominated as T. soudanense, was identical to the remaining two T. violaceum strains, while the second T. soudanense strain, CBS 201.88, had a deletion of 36 bp, as reported in the literature. In the T. tonsurans/T. equinum group, the known C/T SNP was not distributed, as expected, between T. tonsurans and T. equinum strains; only CBS 318.31 had a C SNP, while all remaining 11 strains, including the neotype of T. equinum CBS 270.66, had a T SNP. The sequence of CBS 318.31 was identical to that of the neotype of T. tonsurans CBS 496.48. Trichophyton interdigitale differed from the T. tonsurans/T. equinum group in 9 nucleotides.

### Deconvolution of raw mass spectra.

Results from deconvoluting MS1 spectra employing a proprietary algorithm (A1) and ProSightPC 3.0 (Xtract), followed by processing MS/MS fragment spectra, are summarized in Table 2. Total numbers of identified monoisotopic protein masses are given per replicate, with shared masses per strain, average numbers, standard deviations (SD), and coefficients of variation (CV) representing biological variation. With algorithm A1, the lowest number of monoisotopic masses (52) was observed in T. rubrum CBS 115314 and the highest (259) in T. interdigitale CBS 119447. Standard deviations ranged between 3 and 53 in T. rubrum CBS 202.88 and T. violaceum CBS 120316, respectively. The CV was above 20% for five strains, being the lowest (1%) in T. rubrum CBS 118892 and the highest (27%) in T. equinum CBS 112198.

**TABLE 2:** Numbers of obtained monoisotopic molecular masses by deconvolution of the MS1 spectra with algorithm A1 and ProSightPC 3.0^*a*^

Strain	Algorithm A1							ProSightPC 3.0							Shared mass by ProteinCenter
	Total no. of identified proteins			Statistics			Shared mass	Total no. of identified proteins			Statistics			Shared mass	
	rep 1	rep 2	rep 3	Avg	SD	CV		rep 1	rep 2	rep 3	Avg	SD	CV		
T. rubrum CBS 115314	140	197	130	156	36	23	52	306	552	320	393	138	35	153	16
T. rubrum CBS 100084	182	214	162	186	26	14	88	392	373	320	362	37	10	195	21
T. rubrum CBS 100238	276	314	299	296	19	6	170	629	647	727	668	52	8	457	50
T. rubrum CBS 288.86	417	433	340	397	50	13	205	785	818	749	784	35	4	486	51
T. rubrum CBS 202.88	228	233	227	229	3	1	130	464	644	551	553	90	16	432	45
T. rubrum CBS 118892	346	311	367	341	28	8	195	675	655	810	713	84	12	523	53
T. violaceum CBS 120320	287	190	249	242	49	20	95	395	385	451	410	36	9	304	30
T. violaceum CBS 120316	218	323	277	273	53	19	147	383	591	536	503	108	21	309	39
T. violaceum CBS 201.88	264	257	286	269	15	6	160	697	711	664	691	24	3	513	56
T. violaceum CBS 452.61	394	380	338	371	29	8	218	535	775	680	663	121	18	313	32
T. tonsurans CBS 285.30	336	282	305	308	27	9	187	535	547	419	500	71	14	277	43
T. tonsurans CBS 318.31	153	170	126	150	22	15	81	290	313	227	277	45	16	202	28
T. tonsurans CBS 856.71	124	116	80	107	23	22	57	137	193	159	163	28	17	126	22
T. tonsurans CBS 127.97	345	327	337	336	9	3	193	456	441	454	450	8	2	340	50
T. tonsurans CBS 109033	322	350	308	327	21	7	148	459	340	607	469	134	29	241	43
T. tonsurans CBS 112186	313	251	225	263	45	17	119	494	473	353	440	76	17	262	42
T. equinum CBS 270.66	134	185	205	175	37	21	86	333	448	385	389	58	15	279	46
T. equinum CBS 634.82	274	313	262	283	27	9	162	297	399	290	329	61	19	231	32
T. equinum CBS 100080	382	396	365	381	16	4	222	479	550	648	559	85	15	329	62
T. equinum CBS 112188	204	256	265	242	33	14	116	486	483	523	497	22	4	325	48
T. equinum CBS 112193	184	225	177	195	26	13	103	368	434	350	384	44	12	266	73
T. equinum CBS 112198	242	145	243	210	56	27	91	420	387	421	409	19	5	300	29
T. interdigitale CBS 119447	395	387	450	411	34	8	259	683	612	697	664	46	7	474	71
T. interdigitale CBS 120318	305	304	292	300	7	2	165	605	568	497	557	55	10	369	54

a Variation between shared masses between all replicates for ProSight/ProteinCenter data processing, with results from different E value/homology level cutoff criteria (see Materials and Methods). rep, replicate.

Deconvolution employing ProSightPC 3.0 (Xtract) rendered the lowest number of protein masses (126) in T. tonsurans CBS 856.71 and the highest (523) in T. rubrum CBS 118892. Standard deviations ranged between 8 in T. tonsurans CBS 127.97 and 38 in T. rubrum CBS 115314. The CV was 3% in T. violaceum CBS 201.88 and was highest, at 35%, in T. rubrum CBS 115314. ProteinCenter analysis of protein masses obtained by ProSightPC 3.0 resulted in a total of 413 proteins present in at least one replicate of one of the 24 strains at 100% sequence homology. The number of identified proteins at the 100% homology level is given in Table 2 after postprocessing of initial ProSight results. The list of these proteins is given in Table S1 in the supplemental material. In all subsequent analyses, only deconvoluted masses (algorithm A1) and/or identified proteins (ProSightPC 3.0) present in all three biological replicates were used.

### Strain classification and species prediction.

Monoisotopic masses obtained by A1 were further analyzed in order to select unique masses per strain, to classify the strains, and to predict species affiliations. Strain classification performed on all replicates with four prediction runs is given in Table S2. The highest classification accuracy (Table 3) is achieved with a score of one, while a no call (i.e., no classification) is defined as zero. The results indicate that all strains affiliated with Trichophyton rubrum, T. violaceum, and T. interdigitale were correctly classified with a classification accuracy of 1. In the Trichophyton tonsurans/T. equinum group, four strains, CBS 318.31, CBS 285.30, CBS 100080, and CBS 865.71, were correctly identified with the maximum classification accuracy. The remaining eight strains in this group were identified with a classification accuracy ranging from 0.42 to 0.92. Unique masses per strain were those masses present in all three replicates of a given strain and absent from all replicates from the remaining 24 strains.

**TABLE 3:** Strain classification obtained by algorithm A2

Trichophyton strain^*a*^	CA^*b*^ (A2)	Unique masses per strain^*c*^ (A1)
T. rubrum CBS 115314	1	(1) 22,241.054
T. rubrum CBS 100084 (T)	1	(4) 18,812.048, 10,706.868, 7,384.616, 7,983.656
T. rubrum CBS 100238	1	(6) 7,480.315, 7,975.984, 22,046.173, 6,201.287, 21,707.120, 7,309.791
T. rubrum CBS 288.86	1	(8) 16,934.943, 9,368.722, 7,881.010, 11,873.910, 18,185.271, 7,779.975, 19,733.724, 11,861.896
T. rubrum CBS 202.88	1	(3) 5,437.895, 8,037.319, 5,227.340
T. rubrum CBS 118892	1	(3) 5,209.149, 12,397.609, 5,338.210
T. violaceum CBS 120320	1	(8) 9,852.837, 13,331.557, 5,301.844, 9,865.257, 5,073.543, 7,874.989, 10,851.584, 7,281.114
T. violaceum CBS 120316	1	(7) 6,067.292, 6,198.456, 7,139.672, 19,264.358, 5,147.600, 5,251.738, 12,669.770
T. violaceum CBS 201.88	1	(6) 6,660.335, 8,404.081, 9,832.479, 7,433.682, 9,363.639, 12,924.041
T. violaceum CBS 452.61	1	(7) 5,522.724, 12,042.842, 6,582.882, 6,500.904, 7,781.361, 20794.42548, 12339.29146
T. tonsurans CBS 285.30 (T)	1	(11) 11,388.283, 10,124.535, 23,933.015, 19,316.943, 9,897.097, 13,636.961, 5,183.505, 10,010.170, 15,833.425, 8,114.057, 20915.14524
T. tonsurans CBS 318.31 (T)	1	(5) 6,698.344, 8,927.547, 5,271.958, 7,300.539, 7,523.449
T. tonsurans CBS 856.71	1	(17) 5,169.601, 5,650.649, 5,918.855, 5,934.868, 6,686.769, 6,702.530, 7,005.805, 7,808.024, 8,007.802, 8,148.177, 8,199.929, 8,560.452, 8,915.246, 9,659.657, 9,717.965, 13,008.799, 17,112.085
T. tonsurans CBS 127.97	0.42	(1) 9,945.362
T. tonsurans CBS 109033	0.42	
T. tonsurans CBS 112186	0.92	
T. equinum CBS 270.66 (NT)	0.42	
T. equinum CBS 634.82	0.83	
T. equinum CBS 100080 (T)	1	(11) 11,196.118, 6,496.162, 14,082.605, 5,679.384, 7,750.926, 14,327.736, 6,955.962, 13,134.323, 7,118.017, 6,398.192, 6,105.138
T. equinum CBS 112188	0.67	
T. equinum CBS 112193	0.75	
T. equinum CBS 112198	0.67	
T. interdigitale CBS 119447	1	(16) 9,770.501, 9,659.784, 9,282.621, 5,373.858, 17,020.294, 11,145.854, 28,820.684, 10,022.215, 21,168.266, 21,271.700, 11,174.914, 19,542.778, 19,529.736, 21,354.18,9,908.134, 7,323.754
T. interdigitale CBS 120318	1	(7) 5,065.410, 5,091.356, 5,316.596, 6,488.034, 9,175.903, 13,247.418, 19,206.537

a T, type; NT, neotype.

b CA, coefficient of accuracy.

c The values in parentheses are the numbers of unique masses per strain (in Da).

Species prediction was performed using two independent analyses, each applying one or two reference strains (types or randomly chosen strains) for each cluster (Table 4). While selection of a single reference resulted in inconsistent species calls for both species complexes, adding another strain to the classifier improved classification accuracy by assessing proteome variability. With minor ambiguities, all strains in the T. rubrum complex were correctly identified. In the T. tonsurans complex, one reference-based prediction yielded a random spread of T. tonsurans and T. equinum calls. Addition of a second reference strain assigned only CBS 318.31 and CBS 285.30 to T. tonsurans, while the other 10 strains were assigned to T. equinum (exception in one call for CBS 109033; Table S2).

**TABLE 4:**
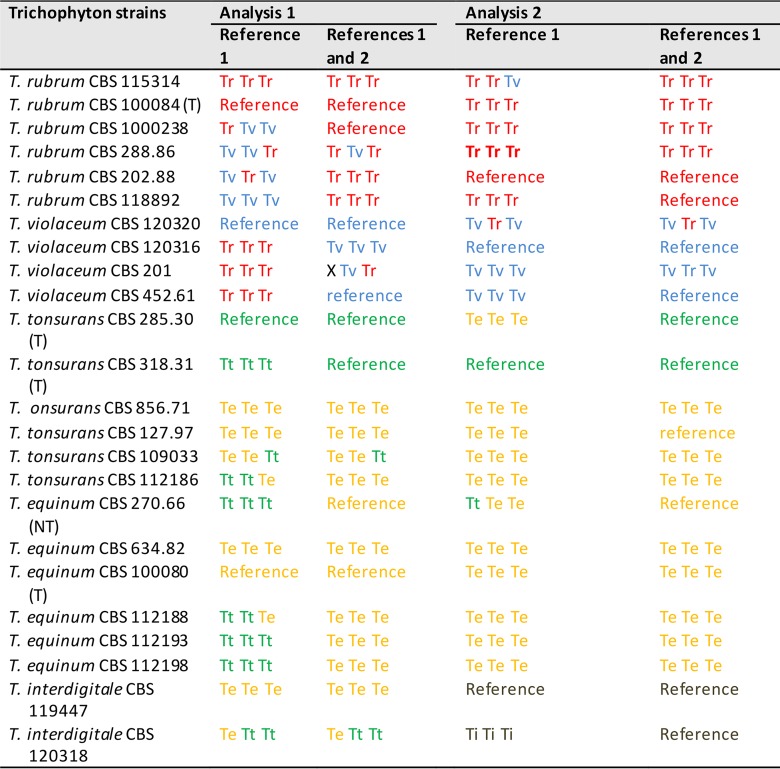
Species prediction of individual Trichophyton strains and the references employed^*a*^

a References are individual mass spectra used relative to the taxon name identified by ITS sequencing. For analysis 1, types, neotypes, and/or randomly chosen strains were used as reference strains. For analysis 2, central strains with the highest number of shared masses (clustering analysis) were used as reference strains. T, type; NT, neotype; Tr, T. rubrum; Tv, T. violaceum; Tt, T. tonsurans; Te, T. equinum; Ti, T. interdigitale; X, no call. Colors visualize taxa.

A second analysis was performed with strains in each species with the highest number of shared masses. The references for T. rubrum, CBS 202.88 and CBS 118892, identified correctly the remaining T. rubrum strains, while in T. violaceum addition of a second reference strain, CBS 452.61, to CBS 120316 did not improve the outcome. Species identifications for all strains primarily identified as T. equinum resulted consistently in T. equinum but never T. tonsurans. Strains CBS 318.31 and CBS 285.30 were consistently assigned as T. tonsurans. The T. interdigitale strains matched each other in the second analysis.

### Selection of unique protein markers.

Unique masses selected by one of the two or both algorithms, A1 and ProSightPC 3.0, are given in Table 5. Homologous proteins in other dermatophytes were matched using 80% for global and 100% for stringent sequence similarity filtering. In the T. rubrum group, both algorithms identified two out of six unique monoisotopic masses. Mass 6,391.358 was identified as hypothetical protein H100_08464 from Trichophyton rubrum MR850 (UniProt entry A0A022T914). Homology search in ProteinCenter revealed this protein in T. equinum, T. interdigitale, and Microsporum gypseum (Nannizzia gypsea), with a monoisotopic mass of 6,419.364 and one amino acid substitution. A second mass of 13,490.093 was identified as V-type ATPase G subunit from Trichophyton rubrum CBS 100081 (UniProt entry A0A022VE64). Homologs were found in T. interdigitale and T. tonsurans with monoisotopic masses of 13,476.077 and 13,480.072, respectively. Four other protein masses identified with ProSightPC 3.0 only were 7,553.051, identified as 40S ribosomal protein S28 from Trichophyton rubrum CBS 100081 (UniProt entry A0A022UXP6), 7,869.165, identified as hypothetical protein H106_04186 from Trichophyton rubrum CBS 735.88 (UniProt entry A0A028JNY6), 10,476.424, identified as hypothetical protein H102_06602 from Trichophyton rubrum CBS 100081 (UniProt entry A0A022UYS5), and 10,974.876, identified as hypothetical protein H107_03773 from Trichophyton rubrum CBS 202.88 (UniProt entry A0A023AI34).

**TABLE 5:** Unique masses per group and per species

Group/species	Unique masses^*a*^
T. rubrum/T. violaceum	(6) 6,391.358*, 13,490.093*, 7,553.051, 7,869.165, 10,476.424, 10,974.876
T. tonsurans/T. equinum/T. interdigitale	(2) 7,883.18*, 10,450.732*
T. tonsurans/T. equinum	(3) 7,906.81*, 8,787.278, 11,464.894
T. equinum/T. interdigitale	(3) 12,993.799, 15,859.410, 15,888.449
T. interdigitale	(5) 6,961.324, 9,014.567, 9,254.624, 9,481.539, 15,829.423
T. tonsurans	(2) 13,152.935, 17,474.951*

a Unique masses were identified by proprietary algorithm 1 and/or ProSightPC 3.0. Proteins marked with an asterisk were identified by both algorithms. The values in parentheses are the numbers of unique masses per strain (in Da).

In the T. tonsurans group (with T. interdigitale as species parameter), two unique masses were found and identified. One was a hypothetical protein, TEQG_02912, from Trichophyton equinum CBS 127.97 (UniProt entry F2PPR0) with a mass of 7,883.18. This protein differs from its counterpart in the T. rubrum group (7,869.165) by one amino acid. The second one was hypothetical protein TEQG_00161, with a mass of 10,450.372, found in T. equinum CBS 127.97 (UniProt entry F2PGT9), differing from the T. rubrum protein (mass of 10,476.424) by having one extra amino acid.

The T. tonsurans group (without T. interdigitale) had three unique masses, of which both algorithms identified mass 7,906.81 as hypothetical protein TEQG_01010 from Trichophyton equinum CBS 127.97 (UniProt entry F2PJA2). The two other monoisotopic masses, 8,787.278 and 11,464.894, could not be identified by ProSightPC 3.0. Trichophyton equinum and T. interdigitale share three masses, 12,993.799, 15,859.411, and 15,888.449. There were no entries for these masses in ProSightPC 3.0. In contrast, T. tonsurans did not have masses in common with T. interdigitale, implying that T. interdigitale likely is closer phylogenetically to T. equinum than to T. tonsurans.

At the species level, no unique masses were found defining T. rubrum, T. violaceum, T. equinum, or T. tonsurans. In contrast, T. interdigitale had five unique masses (6,961.324, 9,014.567, 9,254.624, 9,481.539, and 15,829.423), none of which could be matched to any protein sequence predicted from the corresponding genomes. For T. tonsurans, as derived from the second species prediction approach, the two strains CBS 318.31 and CBS 285.30 affiliated with T. tonsurans share two unique masses: 13,152.935, found by algorithm A1, and 17,474.951, found by both algorithms, identified as hypothetical protein TESG_03051 from T. tonsurans CBS 112818 (UniProt entry F2RWA2).

## DISCUSSION

In this study, we evaluated the resolution power of LC-MS/MS as a novel method to delimit clinically relevant filamentous fungi with two groups of dermatophytes, each containing two very closely related species as a model set. Nucleic acid-based approaches, like rDNA ITS sequence data, are used as a gold standard, as this gene is judged to be optimal for dermatophyte diagnostics (3).

Separation of species within both groups is problematic and highly controversial. On the basis of molecular data, Trichophyton tonsurans (on humans) and T. equinum (on horses) had been regarded as synonyms (15). Matruchot and Dassonville (18) already reported transmission from horse to human in their original description of T. equinum. In the present study, two of the analyzed strains were used that had been transmitted from horse to human: CBS 127.97 (19) and CBS 270.66 (20). Woodgyer (21) distinguished the species by a T/C SNP in ITS1 (C nucleotide in T. tonsurans, T nucleotide in T. equinum), and Chollet et al. (22) listed some phenotypic differences. We were unable to find correspondence between these criteria among our strains using the intact protein-based approach described here. All but one strain (CBS 318.31) listed as T. tonsurans had the T. equinum-associated T nucleotide, which was also present in all six T. equinum strains. To verify the validity of this SNP in a larger data set, we randomly selected 64 strains from the CBS collection (data not shown). All T. equinum strains from horse had a T nucleotide, but 35% of the T. tonsurans strains from humans had the same T nucleotide (data not shown). De Hoog et al. (3) were also unable to distinguish the two species using additional genes. More detailed patient and phenotypic information is necessary to establish whether T. equinum is a separate species at all. Using MALDI-TOF, Nenoff et al. (8) and De Respinis et al. (4) distinguished T. tonsurans and T. equinum, but the authors did not present the grounds on which they denominated the strains as T. tonsurans or T. equinum. In our study, discrimination at strain level resulted in identification of 4 out of 12 strains of the T. tonsurans lineage. The remaining eight strains formed two clusters with overlapping proteomes: cluster 1 with CBS 642.82, CBS 127.97, and CBS 109033, and cluster 2 with two subclusters, CBS 270.66 (overlapping with 112188), CBS 112198 (overlapping with 112186), and CBS 112193 (see Table S2 in the supplemental material). In Fig. 1, these two clusters would be placed in the intersection of the two species clouds. Note that both clusters have strains isolated from both humans and horses, which contradicts the hypothesis of host-based distinction in the two species. In the species prediction analysis (Table 4), only CBS 318.31 and CBS 285.30 were affiliated with T. tonsurans, which is not in concordance with a T/C SNP for CBS 285.30. So far, typing strains within the T. tonsurans/T. equinum species complex appears to be challenging (pending method improvements) due to insufficiently resolved taxonomic definitions of known reference strains, which is likely due to conspecificity.

In MALDI-TOF analyses of De Respinis et al. (4), some T. tonsurans spectra were misidentified as T. interdigitale. Calderaro et al. (23) noted the same misidentification before the supplementation to Bruker's BioTyper database. With nine nucleotides of difference in the ITS region, T. interdigitale should be easily distinguishable from the T. tonsurans/T. equinum complex. Separation was confirmed in all our analyses, with strain classification accuracies of 1, five unique species masses (Table 5), and their clustering as a distinct group in species prediction analysis (Table 4).

Analyses of the T. rubrum group were in concordance with previous findings. According to Gräser et al. (14), the T. rubrum group comprises only two anthropophilic species, T. rubrum and T. violaceum, the latter species with T. soudanense as a probable mutant and prevalently causing tinea capitis. Trichophyton violaceum is endemic to Africa (14, 24), while T. rubrum is cosmopolitan. Microsatellite analysis has revealed that T. violaceum is more variable than T. rubrum, with some strains being closer to T. rubrum than the others (25). Trichophyton rubrum and T. violaceum are morphologically very different but are similar in their DNA profiles. Our analyzed strains differed in four positions in ITS (data not shown). MALDI-TOF analyses frequently proved to be unable to separate the two species (4, 7, 8, 11). Summarized misidentifications and/or unreliable identifications of T. tonsurans (misidentified with T. rubrum and *vice versa*), T. violaceum, and T. soudanense were recently reported by Sanguinetti and Posteraro (26). In the newest evaluation study of the Vitek v3.0 system for the identification of filamentous fungi (27), Trichophyton species were regarded as particularly problematic, with T. interdigitale, T. tonsurans, and T. violaceum having success percentages of 97%, 91%, and 41%, respectively, in the first attempt.

With LC-MS/MS, discrimination at the strain level was achieved with all six T. rubrum and four T. violaceum strains classified with a classification analysis of 1. In this analysis, optimal species association was achieved with CBS 118892 as the reference for T. rubrum and CBS 120316 as the reference for T. violaceum. Notably, taxonomic types may be located eccentrically in the species cloud and thus provide less optimal results. Our analysis showed that T. violaceum strain CBS 120320 shares some features with T. rubrum (with one call as T. rubrum) (Table 4). Both strains denominated in the CBS collection as T. soudanense, CBS 452.61 and CBS 201.88, were affiliated with T. violaceum, fitting the ITS data. Interestingly, the only strain with a 36-bp deletion, CBS 201.88, had one nonsense and one T. violaceum call in the first species prediction analysis (Table 4).

### Conclusions.

Whole-protein top-down LC-MS/MS analysis has significant diagnostic potential because of its analytical performance level being higher than that of MALDI-TOF, particularly below the species level, i.e., at the lineage or strain level. The accurate detection of protein masses, separation of high numbers of individual proteins, and detection of single-amino-acid exchanges are responsible for the high performance. The proprietary Thermo Scientific algorithms A2, A3, and A4 showed a potential to recognize individual strains that can be applied in epidemics or outbreak scenarios. However, detailed studies are required, since the choice of reference strains is crucial for appropriate species affiliation, as routine selection of taxonomic types may not provide optimal results. Species limits and species variability in dermatophytes, which were classically distinguished on the basis of clinical and phenotypic criteria, have to be newly defined in order to develop reliable and predictive taxonomy and meaningful diagnostics tools.

## Supplementary Material

Supplemental material
