# Supplementary material for: Ultra-High-Resolution Mass Spectrometry for Identification of Closely Related Dermatophytes with Different Clinical Predilections
Source: J Clin Microbiol. 2018 Jun 25;56(7):e00102-18. doi: 10.1128/JCM.00102-18 (PMC6018324; doi:10.1128/JCM.00102-18)
Supplement: Supplemental material [file supp_56_7_e00102-18__index.html]

Supplemental material 

# Ultra-High-Resolution Mass Spectrometry for Identification of Closely Related Dermatophytes with Different Clinical Predilections

## Supplemental material

- Supplemental file 1 -

  Table S1 (A list of identified proteins found in at least one replicate in at least one of the 24 strains)

  PDF, 422K
- Supplemental file 2 -

  Table S2 (Strain classification by algorithm A2)

  PDF, 56K
