# Supplementary material for: Ultra-High-Resolution Mass Spectrometry for Identification of Closely Related Dermatophytes with Different Clinical Predilections
Source: J Clin Microbiol. 2018 Jun 25;56(7):e00102-18. doi: 10.1128/JCM.00102-18 (PMC6018324; doi:10.1128/JCM.00102-18)
Supplement: Supplemental material [file JCM.00102-18_zjm999095997s1.pdf]

Table ST1. A list of identified proteins found in at least one replicate in at least one of the 24 strains.

No.	Monoisotopic Mass	Description	Taxon	Uniprot entry
1	2858.46	Full=83 kDa hypersensitivity protein; Short=Protein IV	Trichophyton tonsurans	P80514
2	5024.624	hypothetical protein H101_08178, partial	Trichophyton interdigitale H6	A0A022U3H1
3	5950.082	hypothetical protein TRV_01005	Trichophyton verrucosum HKI 0517	D4D1R2
4	5976.042	hypothetical protein TERG_03909	Trichophyton rubrum CBS 118892	F2SPB0
5	6082.104	hypothetical protein H109_03307	Trichophyton interdigitale MR816	A0A059JAQ7
6	6111.297	TOM complex component Tom7	Trichophyton equinum CBS 127.97	F2PV03
7	6249.552	60S ribosomal protein L39	Trichophyton soudanense CBS 452.61	A0A022Y8Q8
8	6374.187	hypothetical protein H104_04119	Trichophyton rubrum CBS 289.86	A0A022WZD0
**9**	**6391.358**	**hypothetical protein H100_08464**	**Trichophyton rubrum MR850**	**A0A022T914**
10	6419.364	hypothetical protein TEQG_08592	Trichophyton equinum CBS 127.97	F2PIW8
11	6431.245	hypothetical protein TERG_03909	Trichophyton rubrum CBS 118892	F2SPB0
12	6491.121	hypothetical protein MGYG_03527	Nannizzia gypsea CBS 118893	E4USF6
13	6642.338	40S ribosomal protein S29	Trichophyton rubrum MR1459	A0A022ZCH4
14	6657.771	ribosomal protein L33	Trichophyton rubrum CBS 100081	A0A022V366
15	6673.766	mitochondrial 54S ribosomal protein YmL39	Arthroderma otae CBS 113480	C5FQE6
16	6823.45	hypothetical protein TERG_11971	Trichophyton rubrum CBS 118892	A0A080WK51
17	6934.826	hypothetical protein H102_01912	Trichophyton rubrum CBS 100081	A0A022VEY7
18	7025.632	hypothetical protein MGYG_08779	Nannizzia gypsea CBS 118893	E4V6Z3
19	7164.456	hypothetical protein H103_05209	Trichophyton rubrum CBS 288.86	A0A022W0B9
20	7294.897	H/ACA ribonucleoprotein complex subunit 3	Trichophyton interdigitale MR816	A0A059JAQ0
21	7310.892	H/ACA ribonucleoprotein complex subunit 3	Arthroderma otae CBS 113480	C5FVJ5
22	7320.715	hypothetical protein TESG_03327	Trichophyton tonsurans CBS 112818	F2RX14
23	7334.73	hypothetical protein H109_04377	Trichophyton interdigitale MR816	A0A059J8G2
24	7336.71	hypothetical protein H106_05993	Trichophyton rubrum CBS 735.88	A0A028JJJ7
25	7350.611	hypothetical protein TESG_05851	Trichophyton tonsurans CBS 112818	F2S4H9

26	7370.694	hypothetical protein H105_06170	Trichophyton soudanense CBS 452.61	A0A022XMJ5
27	7420.67	hypothetical protein H112_04983	Trichophyton rubrum D6	A0A059JZF8
28	7422.632	hypothetical protein TEQG_04554	Trichophyton equinum CBS 127.97	F2PUH7
29	7471.856	Mitotic-spindle organizing protein 1	Trichophyton interdigitale H6	A0A022U716
30	7485.872	hypothetical protein MGYG_08659	Nannizzia gypsea CBS 118893	E4V6L8
31	7499.887	Mitotic-spindle organizing protein 1	Trichophyton rubrum CBS 289.86	A0A022WVV9
32	7549.887	40S ribosomal protein S29	Trichophyton equinum CBS 127.97	F2PHG7
33	**7553.051**	**40S ribosomal protein S28**	**Trichophyton rubrum CBS 100081**	**A0A022UXP6**
34	7569.045	40S ribosomal protein S28	Trichophyton interdigitale MR816	A0A059J6B3
35	7593.88	hypothetical protein H109_01378	Trichophyton interdigitale MR816	A0A059JG54
36	7647.822	hypothetical protein TEQG_05605	Trichophyton equinum CBS 127.97	F2PXI9
37	7690.933	hypothetical protein H107_02000	Trichophyton rubrum CBS 202.88	A0A023AQ26
38	7695.827	hypothetical protein H103_00600	Trichophyton rubrum CBS 288.86	A0A022WGL4
39	7708.907	hypothetical protein TEQG_00547	Trichophyton equinum CBS 127.97	F2PHT5
40	7755.104	protein translocase SEC61 complex gamma subunit	Trichophyton rubrum CBS 202.88	A0A023A6D6
41	7755.104	conserved hypothetical protein	Arthroderma otae CBS 113480	C5FZC5
42	7776.126	Non-histone chromosomal protein 6	Trichophyton interdigitale H6	A0A022U2R8
43	7828.147	hypothetical protein MGYG_08266	Nannizzia gypsea CBS 118893	E4V672
44	7831.146	hypothetical protein H100_03016	Trichophyton rubrum MR850	A0A022TSI8
45	**7869.165**	**hypothetical protein H106_04186**	**Trichophyton rubrum CBS 735.88**	**A0A028JNY6**
46	7876.799	hypothetical protein H109_05871	Trichophyton interdigitale MR816	A0A059J412
47	**7883.18**	**hypothetical protein TEQG_02912**	**Trichophyton equinum CBS 127.97**	**F2PPR0**
48	**7906.81**	**hypothetical protein TEQG_01010**	**Trichophyton equinum CBS 127.97**	**F2PJA2**
49	7911.063	hypothetical protein H105_06878	Trichophyton soudanense CBS 452.61	A0A022XJV4
50	7936.903	hypothetical protein H100_08883	Trichophyton rubrum MR850	A0A022T7A3
51	7995.084	hypothetical protein H103_06623	Trichophyton rubrum CBS 288.86	A0A022VV02
52	8132.933	hypothetical protein H101_07725	Trichophyton interdigitale H6	A0A022U460
53	8147.908	hypothetical protein TERG_02953	Trichophyton rubrum CBS 118892	F2SLT9
54	8262.385	Nop10 family nucleolar RNA-binding protein	Trichophyton tonsurans CBS 112818	F2RN39
55	8349.431	conserved hypothetical protein	Arthroderma otae CBS 113480	C5G0N6
56	8500.393	hypothetical protein H106_03055	Trichophyton rubrum CBS 735.88	A0A028JU11

57	8526.187	metallothionein-I transcription activator	Trichophyton rubrum CBS 118892	F2SXL9
58	8526.187	metallothionein-I transcription activator	Trichophyton tonsurans CBS 112818	F2S805
59	8546.188	hypothetical protein H105_08314	Trichophyton soudanense CBS 452.61	A0A022XEU8
60	8560.203	hypothetical protein H113_08378	Trichophyton rubrum MR1459	A0A022Z5R6
61	8655.356	hypothetical protein TERG_08213	Trichophyton rubrum CBS 118892	F2T075
62	8677.222	hypothetical protein TEQG_05411	Trichophyton equinum CBS 127.97	F2PWY9
63	8690.217	hypothetical protein H109_00201	Trichophyton interdigitale MR816	A0A059JKQ0
64	8704.232	hypothetical protein H102_00282	Trichophyton rubrum CBS 100081	A0A022VLB6
65	8750.411	ribosomal protein S21e	Trichophyton tonsurans CBS 112818	F2S3S0
66	8784.538	hypothetical protein TESG_08443	Trichophyton tonsurans CBS 112818	F2RYS1
67	8840.68	hypothetical protein TESG_06208	Trichophyton tonsurans CBS 112818	F2S5J5
68	8840.694	Small nuclear ribonucleoprotein SmG	Trichophyton rubrum CBS 118892	F2SIV7
69	8908.57	40S ribosomal protein S27	Trichophyton rubrum CBS 289.86	A0A022WNF6
70	8918.592	40S ribosomal protein S27	Arthroderma otae CBS 113480	C5FZK4
71	8963.868	60S ribosomal protein, partial	Trichophyton rubrum MR1459	A0A022ZRD6
72	9011.974	hypothetical protein H113_05862	Trichophyton rubrum MR1459	A0A022ZFB8
73	9067.354	hypothetical protein H104_00893	Trichophyton rubrum CBS 289.86	A0A022XAF9
74	9130.469	hypothetical protein H106_08183	Trichophyton rubrum CBS 735.88	A0A028JB47
75	9140.068	hypothetical protein H103_05821	Trichophyton rubrum CBS 288.86	A0A022VZ71
76	9232.089	hypothetical protein H110_06181	Trichophyton rubrum MR1448	A0A022YGY3
77	9244.486	hypothetical protein H109_03933	Trichophyton interdigitale MR816	A0A059J8L9
78	9246.141	60s ribosomal protein l38	Trichophyton equinum CBS 127.97	F2PW41
79	9256.941	60S ribosomal protein	Trichophyton rubrum MR1459	A0A022ZSJ9
80	9348.979	Cytochrome b5	Trichophyton equinum CBS 127.97	F2PP14
81	9350.94	cytochrome b5, putative	Trichophyton benhamiae CBS 112371	D4AU60
82	9376.985	hypothetical protein H101_07638	Trichophyton interdigitale H6	A0A022U4D1
83	9377.01	cytochrome b5, putative	Trichophyton verrucosum HKI 0517	D4DE89
84	9561.734	hypothetical protein H110_00692	Trichophyton rubrum MR1448	A0A022Z154
85	9577.782	hypothetical protein TEQG_08164	Trichophyton equinum CBS 127.97	F2Q4Z7
86	9583.833	40S ribosomal protein S21	Trichophyton rubrum CBS 735.88	A0A028JYC2
87	9591.983	hypothetical protein H100_06193	Trichophyton rubrum MR850	A0A022TGZ4

88	9635.788	Heat shock protein Awh11, putative	Trichophyton verrucosum HKI 0517	D4D992
89	9635.788	Heat shock protein Awh11, putative	Trichophyton benhamiae CBS 112371	D4ATB9
90	9642.968	hypothetical protein H112_01581	Trichophyton rubrum D6	A0A059KBL2
91	9665.799	hypothetical protein H109_06473	Trichophyton interdigitale MR816	A0A059J136
92	9681.793	hypothetical protein H112_08565	Trichophyton rubrum D6	A0A059JLR6
93	9811.563	conidiation-specific protein Con-10, putative	Trichophyton verrucosum HKI 0517	D4DAE6
94	9839.594	conidiation-specific protein Con-10, putative	Trichophyton benhamiae CBS 112371	D4AUM8
95	9853.717	conserved hypothetical protein	Arthroderma otae CBS 113480	C5FW50
96	9859.529	Conidiation-specific protein 10	Trichophyton interdigitale MR816	A0A059J9Z7
97	9873.545	hypothetical protein TEQG_08697	Trichophyton equinum CBS 127.97	F2PUC7
98	9876.732	hypothetical protein TRV_05378	Trichophyton verrucosum HKI 0517	D4DE14
99	9890.062	hypothetical protein H104_03541	Trichophyton rubrum CBS 289.86	A0A022X1B9
100	9898.956	hypothetical protein TESG_00720	Trichophyton tonsurans CBS 112818	F2RPB1
101	9906.743	hypothetical protein H103_04223	Trichophyton rubrum CBS 288.86	A0A022W3V7
102	9934.737	hypothetical protein H109_03021	Trichophyton interdigitale MR816	A0A059JBF5
103	10101.517	hypothetical protein H109_02823	Trichophyton interdigitale MR816	A0A059JCG1
104	10105.512	hypothetical protein H102_03915	Trichophyton rubrum CBS 100081	A0A022V8Q4
105	10115.532	hypothetical protein TESG_07549	Trichophyton tonsurans CBS 112818	F2S9I3
106	10119.491	hypothetical protein MGYG_03413	Nannizzia gypsea CBS 118893	E4UNF9
107	10145.306	40S ribosomal protein S27	Nannizzia gypsea CBS 118893	E4UWP1
108	10189.469	60S ribosomal protein L43	Nannizzia gypsea CBS 118893	E4V424
109	10205.464	60S ribosomal protein L43	Trichophyton rubrum D6	A0A059K219
110	10219.479	60S ribosomal protein L43	Trichophyton interdigitale MR816	A0A059JBX3
111	10327.038	hypothetical protein H107_06055	Trichophyton rubrum CBS 202.88	A0A023A940
112	10371.184	hypothetical protein TEQG_03178	Trichophyton equinum CBS 127.97	F2PQH7
113	10387.179	hypothetical protein H103_04689	Trichophyton rubrum CBS 288.86	A0A022W1W8
114	10425.134	hypothetical protein H104_08266	Trichophyton rubrum CBS 289.86	A0A022WKH6
115	10425.134	NADH-ubiquinone oxidoreductase B18 subunit	Trichophyton tonsurans CBS 112818	F2S0M1
116	**10450.372**	**hypothetical protein TEQG_00161**	**Trichophyton equinum CBS 127.97**	**F2PGT9**
117	**10476.424**	**hypothetical protein H102_06602**	**Trichophyton rubrum CBS 100081**	**A0A022UYS5**

118	10478.379	hypothetical protein TRV_00578	Trichophyton verrucosum HKI 0517	D4D0I3
119	10519.148	ribosomal protein A1	Trichophyton tonsurans CBS 112818	F2RZP6
120	10586.893	40S ribosomal protein S25	Nannizzia gypsea CBS 118893	E4V2D3
121	10588.872	40S ribosomal protein S25	Arthroderma otae CBS 113480	C5FBE7
122	10616.939	40S ribosomal protein S25	Trichophyton equinum CBS 127.97	F2Q2F3
123	10678.814	LSM domain-containing protein	Nannizzia gypsea CBS 118893	E4V3J8
124	10736.82	hypothetical protein H113_03339	Trichophyton rubrum MR1459	A0A022ZNK2
125	10776.231	NADH:ubiquinone oxidoreductase kDa subunit	Nannizzia gypsea CBS 118893	E5R3T8
126	10782.769	hypothetical protein H107_03434	Trichophyton rubrum CBS 202.88	A0A023AIQ4
127	10818.278	hypothetical protein H112_07904	Trichophyton rubrum D6	A0A059JNV3
128	10838.562	conserved hypothetical protein	Arthroderma otae CBS 113480	C5FUI5
129	10858.644	hypothetical protein H109_07557	Trichophyton interdigitale MR816	A0A059IY00
130	10861.284	NADH:ubiquinone oxidoreductase 11.5kD subunit	Trichophyton tonsurans CBS 112818	F2S2S4
131	10868.487	Ubiquinol-cytochrome c reductase complex	Trichophyton equinum CBS 127.97	F2Q5B3
132	10888.654	hypothetical protein H103_00309	Trichophyton rubrum CBS 288.86	A0A022WG40
133	10907.128	Cytochrome c oxidase polypeptide vib	Nannizzia gypsea CBS 118893	E4UQM5
134	10930.585	glutaredoxin	Trichophyton rubrum CBS 289.86	A0A022WLE1
135	**10974.876**	**hypothetical protein H107_03773**	**Trichophyton rubrum CBS 202.88**	**A0A023AI34**
136	10986.67	glutaredoxin	Trichophyton interdigitale MR816	A0A059J445
137	11002.907	ATP synthase subunit F	Trichophyton tonsurans CBS 112818	F2S384
138	11003.413	hypothetical protein MGYG_01517	Nannizzia gypsea CBS 118893	E5R1A0
139	11003.413	60S acidic ribosomal protein P1	Trichophyton interdigitale MR816	A0A059JID4
140	11004.887	hypothetical protein H101_03632	Trichophyton interdigitale H6	A0A022UFQ5
141	11017.429	hypothetical protein ARB_00018	Trichophyton benhamiae CBS 112371	D4AV09
142	11025.476	hypothetical protein H104_03149	Trichophyton rubrum CBS 289.86	A0A022X2C4
143	11033.424	60S acidic ribosomal protein P1	Arthroderma otae CBS 113480	C5FCM0
144	11044.954	ATP synthase f chain	Arthroderma otae CBS 113480	C5FM10
145	11051.241	hypothetical protein H104_05804	Trichophyton rubrum CBS 289.86	A0A022WSK7
146	11107.405	hypothetical protein TRV_07083	Trichophyton verrucosum HKI 0517	D4DIS2
147	11128.031	hypothetical protein H104_00386	Trichophyton rubrum CBS 289.86	A0A022XBP6
148	11134.417	60S acidic ribosomal protein P2	Trichophyton interdigitale H6	A0A022UDH1

149	11134.417	hypothetical protein ARB_06463	Trichophyton benhamiae CBS 112371	D4AQF6
150	11161.428	60S acidic ribosomal protein P2	Trichophyton soudanense CBS 452.61	A0A022XKK8
151	11164.428	60S acidic ribosomal protein P2	Trichophyton tonsurans CBS 112818	F2RX80
152	11181.035	Mitochondrial heat shock protein Hsp10	Trichophyton benhamiae CBS 112371	D4B2T3
153	11343.367	Histone	Trichophyton rubrum CBS 100081	A0A022V2V7
154	11373.378	Histone	Trichophyton rubrum D6	A0A059JV68
155	11401.486	Cytochrome c oxidase polypeptide vib	Trichophyton equinum CBS 127.97	F2PLP7
156	11413.653	hypothetical protein H109_00222	Trichophyton interdigitale MR816	A0A059JJW1
157	11416.061	60S ribosomal protein	Trichophyton rubrum MR850	A0A022TUU8
158	11446.07	60S ribosomal protein L30	Arthroderma otae CBS 113480	C5FTF5
159	11491.878	Acyl CoA binding protein family protein	Trichophyton benhamiae CBS 112371	D4AZ00
160	11495.801	hypothetical protein H107_03639	Trichophyton rubrum CBS 202.88	A0A023AI70
161	11511.194	hypothetical protein H109_06884	Trichophyton interdigitale MR816	A0A059J064
162	11530.61	hypothetical protein H105_00298	Trichophyton soudanense CBS 452.61	A0A022Y7U0
163	11537.884	Acyl CoA binding protein family	Trichophyton verrucosum HKI 0517	D4D796
164	11537.884	hypothetical protein H109_02564	Trichophyton interdigitale MR816	A0A059JD51
165	11553.915	hypothetical protein TEQG_01139	Trichophyton equinum CBS 127.97	F2PJN2
166	11604.74	CipC protein	Arthroderma otae CBS 113480	C5FU43
167	11678.783	eukaryotic translation initiation factor 5A	Trichophyton equinum CBS 127.97	F2PJS6
168	11690.619	60S ribosomal protein L36	Trichophyton rubrum CBS 735.88	A0A028JH03
169	11706.614	60S ribosomal protein L36	Nannizzia gypsea CBS 118893	E4UW88
170	11710.05	hypothetical protein H113_05269	Trichophyton rubrum MR1459	A0A022ZH00
171	11718.65	60S ribosomal protein L36	Trichophyton verrucosum HKI 0517	D4D6R6
172	11727.004	Non-histone chromosomal protein 6	Trichophyton rubrum MR1448	A0A022YXQ9
173	11771.03	Nucleosome binding protein	Trichophyton tonsurans CBS 112818	F2S7D0
174	11778.896	hypothetical protein H110_08751	Trichophyton rubrum MR1448	A0A022Y9X9
175	11842.063	hypothetical protein TESG_00838	Trichophyton tonsurans CBS 112818	F2RPQ6
176	11843.046	hypothetical protein H109_06227	Trichophyton interdigitale MR816	A0A059J2X1
177	11849.368	hypothetical protein H101_08083, partial	Trichophyton interdigitale H6	A0A022U338
178	11872.059	NADH-ubiquinone oxidoreductase subunit	Nannizzia gypsea CBS 118893	E4UVI5
179	11898.425	60S ribosomal protein L33	Arthroderma otae CBS 113480	C5FQK6

180	11899.141	hypothetical protein H101_01833	Trichophyton interdigitale H6	A0A022UMC2
181	11906.043	hypothetical protein ARB_02394	Trichophyton benhamiae CBS 112371	D4B1R3
182	12009.764	hypothetical protein TRV_02149	Trichophyton verrucosum HKI 0517	D4D4Y1
183	12024.738	hypothetical protein ARB_00575	Trichophyton benhamiae CBS 112371	D4AWK9
184	12087.505	LYR family protein	Trichophyton equinum CBS 127.97	F2PL66
185	12156.671	chaperonin GroS	Nannizzia gypsea CBS 118893	E5R1X1
186	12179.059	hypothetical protein H112_01497	Trichophyton rubrum D6	A0A059KAG9
187	12193.109	cytochrome c	Trichophyton rubrum CBS 118892	A0A080WFY2
188	12297.663	60S ribosomal protein L33-A	Trichophyton rubrum CBS 289.86	A0A022WV96
189	12298.647	60S ribosomal protein L33-A	Trichophyton interdigitale H6	A0A022ULJ8
190	12302.329	hypothetical protein TRV_00851	Trichophyton verrucosum HKI 0517	D4D1A3
191	12335.315	hypothetical protein TEQG_06872	Trichophyton equinum CBS 127.97	F2Q1V3
192	12348.616	chaperonin 10 Kd subunit	Trichophyton tonsurans CBS 112818	F2RWD9
193	12526.289	hypothetical protein TRV_07039	Trichophyton verrucosum HKI 0517	D4DIM9
194	12543.25	hypothetical protein H101_01020	Trichophyton interdigitale H6	A0A022UPB3
195	12661.688	Ribonucleoprotein-associated protein	Trichophyton equinum CBS 127.97	F2PK61
196	12753.907	ribosomal L15	Nannizzia gypsea CBS 118893	E4V4H5
197	12824.541	hypothetical protein TESG_01520	Trichophyton tonsurans CBS 112818	F2RRT2
198	13043.89	translation initiation factor SUI1	Trichophyton rubrum D6	A0A059JSM3
199	13048.586	progesterone binding protein	Nannizzia gypsea CBS 118893	E5R411
200	13075.634	progesterone binding protein	Trichophyton tonsurans CBS 112818	F2RRT7
201	13102.645	hypothetical protein H112_04837	Trichophyton rubrum D6	A0A059K064
202	13143.114	hypothetical protein TESG_02806	Trichophyton tonsurans CBS 112818	F2RVH0
203	13158.933	LEA domain protein	Trichophyton verrucosum HKI 0517	D4D0M9
204	13159.815	hypothetical protein H107_05463	Trichophyton rubrum CBS 202.88	A0A023ACA9
205	13163.717	hypothetical protein H106_00948	Trichophyton rubrum CBS 735.88	A0A028JZH6
206	13216.942	hypothetical protein H101_02845	Trichophyton interdigitale H6	A0A022UIA8
207	13257.254	40S ribosomal protein S20	Trichophyton rubrum MR1448	A0A022YE10
208	13263.806	hypothetical protein TEQG_02091	Trichophyton equinum CBS 127.97	F2PMF7
209	13476.077	V-type ATPase, G subunit	Trichophyton interdigitale H6	A0A022UJR0
210	13480.072	vacuolar ATPase	Trichophyton tonsurans CBS 112818	F2S9B6

211	**13490.093**	**V-type ATPase, G subunit**	**Trichophyton rubrum CBS 100081**	**A0A022VE64**
212	13510.123	hypothetical protein H101_07032	Trichophyton interdigitale H6	A0A022U5Y4
213	13636.525	60S ribosomal protein L34-B	Nannizzia gypsea CBS 118893	E4V0E3
214	13647.541	60S ribosomal protein L34	Arthroderma otae CBS 113480	C5FT72
215	13661.233	small nucleolar ribonucleoprotein SNU13	Arthroderma otae CBS 113480	C5FM70
216	13663.536	hypothetical protein H112_02454	Trichophyton rubrum D6	A0A059K739
217	13686.776	hypothetical protein TESG_00525	Trichophyton tonsurans CBS 112818	F2RNQ9
218	13691.244	Ribonucleoprotein-associated protein	Trichophyton rubrum CBS 288.86	A0A022W4Q7
219	13707.469	50S ribosomal protein L31e	Nannizzia gypsea CBS 118893	E5QZ74
220	13747.761	hypothetical protein TEQG_03107	Trichophyton equinum CBS 127.97	F2PQA7
221	13779.49	ribosomal protein L31e	Trichophyton equinum CBS 127.97	F2PYV1
222	13812.979	hypothetical protein H109_02490	Trichophyton interdigitale MR816	A0A059JDJ1
223	13828.51	hypothetical protein H109_00904	Trichophyton interdigitale MR816	A0A059JIM0
224	13898.295	hypothetical protein H106_08509	Trichophyton rubrum CBS 735.88	A0A028JAS5
225	13907.214	hypothetical protein H104_01247	Trichophyton rubrum CBS 289.86	A0A022X8H5
226	13921.229	Endoribonuclease L-PSP	Trichophyton equinum CBS 127.97	F2PM80
227	13928.306	hypothetical protein H103_08635	Trichophyton rubrum CBS 288.86	A0A022VNL9
228	13940.37	fatty acid-binding protein	Arthroderma otae CBS 113480	C5FKZ1
229	13959.311	fatty acid-binding protein	Trichophyton equinum CBS 127.97	F2PYU9
230	13992.269	NADH-ubiquinone oxidoreductase subunit GRIM-19	Trichophyton equinum CBS 127.97	F2Q1E2
231	14060.652	hypothetical protein TRV_05526	Trichophyton verrucosum HKI 0517	D4DEF9
232	14064.29	NADH-ubiquinone oxidoreductase subunit GRIM-19	Trichophyton tonsurans CBS 112818	F2SAI5
233	14066.281	hypothetical protein TESG_00081	Trichophyton tonsurans CBS 112818	F2RMF9
234	14100.265	hypothetical protein H109_07116	Trichophyton interdigitale MR816	A0A059J096
235	14139.376	Mitochondrial import membrane translocase subunit TIM16	Trichophyton rubrum MR1448	A0A022YBT0
236	14167.642	Histone H2A	Arthroderma otae CBS 113480	C5G085
237	14170.526	hypothetical protein H101_01637	Trichophyton interdigitale H6	A0A022ULZ8
238	14186.376	hypothetical protein MGYG_00561	Nannizzia gypsea CBS 118893	E5R0G1
239	14227.199	conserved eukaryotic protein	Trichophyton benhamiae CBS 112371	D4AY16
240	14255.194	hypothetical protein MGYG_04653	Nannizzia gypsea CBS 118893	E4UVZ0
241	14255.206	hypothetical protein H101_06980	Trichophyton interdigitale H6	A0A022U679

242	14259.429	Prefoldin subunit 6	Trichophyton tonsurans CBS 112818	F2S5M6
243	14262.673	hypothetical protein MGYG_05361	Nannizzia gypsea CBS 118893	E4UVN8
244	14269.246	hypothetical protein H103_06195	Trichophyton rubrum CBS 288.86	A0A022VWA2
245	14283.237	hypothetical protein TEQG_05212	Trichophyton equinum CBS 127.97	F2PW31
246	14288.505	translation factor SUI1	Trichophyton rubrum CBS 118892	F2SH11
247	14309.606	hypothetical protein H101_01399	Trichophyton interdigitale H6	A0A022UNI8
248	14310.821	Histone H2A	Trichophyton rubrum CBS 202.88	A0A023A4J6
249	14312.801	Histone H2A	Trichophyton interdigitale MR816	A0A059J5J3
250	14313.439	hypothetical protein H101_02029	Trichophyton interdigitale H6	A0A022UKK6
251	14317.731	30S ribosomal protein S26e	Nannizzia gypsea CBS 118893	E5R195
252	14327.481	ubiquinol-cytochrome c reductase complex protein	Trichophyton equinum CBS 127.97	F2PMN2
253	14330.727	40S ribosomal protein S26E	Trichophyton rubrum CBS 202.88	A0A023ARS4
254	14349.514	hypothetical protein H102_01220	Trichophyton rubrum CBS 100081	A0A022VIB2
255	14360.97	hypothetical protein TEQG_02759	Trichophyton equinum CBS 127.97	F2PPA8
256	14371.802	Histone H2A	Nannizzia gypsea CBS 118893	E4UXT4
257	14412.652	60S ribosomal protein L22	Trichophyton tonsurans CBS 112818	F2RPK6
258	14415.053	hypothetical protein H112_05223	Trichophyton rubrum D6	A0A059JYN8
259	14490.219	60S ribosomal protein L35	Trichophyton interdigitale H6	A0A022UFE6
260	14497.681	hypothetical protein ARB_01782	Trichophyton benhamiae CBS 112371	D4B011
261	14498.7	hypothetical protein H110_08801	Trichophyton rubrum MR1448	A0A022Y956
262	14514.133	hypothetical protein H105_05254	Trichophyton soudanense CBS 452.61	A0A022XPI4
263	14726.445	hypothetical protein H113_02302	Trichophyton rubrum MR1459	A0A022ZRJ3
264	14729.826	60S ribosomal protein L23	Trichophyton rubrum CBS 100081	A0A022VG47
265	14743.842	60S ribosomal protein L23	Arthroderma otae CBS 113480	C5FSH1
266	14754.014	40S ribosomal protein S22	Arthroderma otae CBS 113480	C5FBG8
267	14768.029	40S ribosomal protein S22	Trichophyton rubrum CBS 288.86	A0A022WGN2
268	14771.492	hypothetical protein ARB_04644	Trichophyton benhamiae CBS 112371	D4AK43
269	14781.514	hypothetical protein TRV_01858	Trichophyton verrucosum HKI 0517	D4D444
270	14872.152	hypothetical protein H100_04861	Trichophyton rubrum MR850	A0A022TL60
271	14892.873	hypothetical protein TEQG_06170	Trichophyton equinum CBS 127.97	F2PZ65
272	14895.521	hypothetical protein TEQG_04466	Trichophyton equinum CBS 127.97	F2PTU3

273	14896.142	hypothetical protein TESG_05979	Trichophyton tonsurans CBS 112818	F2S554
274	14923.526	hypothetical protein H109_01627	Trichophyton interdigitale MR816	A0A059JGI0
275	14953.068	hypothetical protein TERG_08341	Trichophyton rubrum CBS 118892	F2T0K4
276	14961.119	Histone H2B	Nannizzia gypsea CBS 118893	E4UXT5
277	14965.245	hypothetical protein TRV_03415	Trichophyton verrucosum HKI 0517	D4D8H9
278	14991.13	Histone H2B	Arthroderma otae CBS 113480	C5G086
279	14995.257	60S ribosomal protein L26	Trichophyton tonsurans CBS 112818	F2RWJ5
280	14995.257	60S ribosomal protein	Nannizzia gypsea CBS 118893	E5R351
281	15034.321	60S ribosomal protein L26	Arthroderma otae CBS 113480	C5FKB1
282	15126.746	hypothetical protein TESG_04470	Trichophyton tonsurans CBS 112818	F2S0F0
283	15129.692	hypothetical protein H106_08258	Trichophyton rubrum CBS 735.88	A0A028JB85
284	15133.204	Histone H2B	Trichophyton tonsurans CBS 112818	F2RT32
285	15144.739	hypothetical protein H102_08361	Trichophyton rubrum CBS 100081	A0A022UUA8
286	15145.724	hypothetical protein H105_08388	Trichophyton soudanense CBS 452.61	A0A022XFK1
287	15156.738	hypothetical protein H109_05911	Trichophyton interdigitale MR816	A0A059J341
288	15172.733	conserved hypothetical protein	Trichophyton benhamiae CBS 112371	D4AYC5
289	15206.835	hypothetical protein TRV_03257	Trichophyton verrucosum HKI 0517	D4D822
290	15227.739	conserved hypothetical protein	Trichophyton verrucosum HKI 0517	D4D870
291	15234.593	hypothetical protein H107_02338	Trichophyton rubrum CBS 202.88	A0A023ANT4
292	15322.534	hypothetical protein MGYG_07574	Nannizzia gypsea CBS 118893	E4V3J4
293	15350.565	hypothetical protein H109_02391	Trichophyton interdigitale MR816	A0A059JD05
294	15477.104	hypothetical protein H100_03277	Trichophyton rubrum MR850	A0A022TRS4
295	15491.616	Histone H3	Trichophyton rubrum MR850	A0A022TJC5
296	15631.59	hypothetical protein H110_07654	Trichophyton rubrum MR1448	A0A022YDN5
297	15633.568	60S ribosomal protein L27	Arthroderma otae CBS 113480	C5FDL3
298	15635.548	60S ribosomal protein L27-A	Nannizzia gypsea CBS 118893	E5QZY6
299	15661.601	hypothetical protein H101_02193	Trichophyton interdigitale H6	A0A022UKC2
300	15800.748	40S ribosomal protein S23	Arthroderma otae CBS 113480	C5G0P7
301	15809.909	hypothetical protein H109_03069	Trichophyton interdigitale MR816	A0A059JB33
302	15831.905	hypothetical protein H103_04287	Trichophyton rubrum CBS 288.86	A0A022W3L4
303	15836.759	30S ribosomal protein S26e	Nannizzia gypsea CBS 118893	E4V6M9

304	15839.619	40S ribosomal protein S16	Trichophyton soudanense CBS 452.61	A0A022XH64
305	15844.76	Cu,Zn superoxide dismutase	Trichophyton tonsurans CBS 112818	F2RNE8
306	15850.774	40S ribosomal protein S23	Trichophyton interdigitale MR816	A0A059J9K0
307	15918.812	Superoxide dismutase [Cu-Zn]	Trichophyton interdigitale MR816	A0A059J130
308	15922.408	glycine-rich RNA-binding protein	Trichophyton tonsurans CBS 112818	F2S5E4
309	15922.781	Superoxide dismutase [Cu-Zn]	Trichophyton rubrum CBS 735.88	A0A028JNY9
310	15961.467	hypothetical protein H109_07937	Trichophyton interdigitale MR816	A0A059IX40
311	15974.787	Cu Zn superoxide dismutase	Arthroderma otae CBS 113480	C5FII9
312	16067.827	superoxide dismutase	Nannizzia gypsea CBS 118893	E4UUM7
313	16153.848	60S ribosomal protein L27-A	Trichophyton equinum CBS 127.97	F2PW54
314	16275.254	50S ribosomal protein YmL27	Trichophyton tonsurans CBS 112818	F2S4R8
315	16278.686	40S ribosomal protein S17	Nannizzia gypsea CBS 118893	E5R1Q0
316	16286.727	40S ribosomal protein S17	Arthroderma otae CBS 113480	C5FBZ3
317	16312.198	ssDNA binding protein	Trichophyton tonsurans CBS 112818	F2S609
318	16347.112	Nascent polypeptide-associated complex subunit beta	Trichophyton rubrum CBS 118892	F2SBS6
319	16354.245	hypothetical protein H106_02934	Trichophyton rubrum CBS 735.88	A0A028JSG5
320	16423.484	40S ribosomal protein S19	Trichophyton rubrum CBS 289.86	A0A022X3Y1
321	16427.738	CHCH domain-containing protein	Trichophyton equinum CBS 127.97	F2Q5Z1
322	16447.695	Ribosomal protein L28e	Trichophyton tonsurans CBS 112818	F2RYV1
323	16627.598	hypothetical protein H109_05054	Trichophyton interdigitale MR816	A0A059J5P8
324	16730.119	ribosomal protein L14	Trichophyton tonsurans CBS 112818	F2RPU3
325	16801.893	hypothetical protein H109_01920	Trichophyton interdigitale MR816	A0A059JF24
326	16809.25	40S ribosomal protein S13	Arthroderma otae CBS 113480	C5FT17
327	16832.242	40S ribosomal protein	Trichophyton interdigitale MR816	A0A059JES5
328	16846.258	40S ribosomal protein	Trichophyton soudanense CBS 452.61	A0A022Y0M1
329	16871.584	Nucleoside diphosphate kinase A	Trichophyton equinum CBS 127.97	F2PS09
330	16874.234	60S ribosomal protein L14	Arthroderma otae CBS 113480	C5FKR8
331	16875.486	hypothetical protein H113_04719	Trichophyton rubrum MR1459	A0A022ZHM3
332	16875.965	hypothetical protein H113_02692	Trichophyton rubrum MR1459	A0A022ZRK5
333	16885.6	Nucleoside diphosphate kinase	Trichophyton tonsurans CBS 112818	F2RNW0
334	16916.594	Nucleoside diphosphate kinase	Trichophyton soudanense CBS 452.61	A0A022XRI5

335	16924.057	60S ribosomal protein L27a	Trichophyton interdigitale MR816	A0A059J573
336	16952.063	60S ribosomal protein L28	Trichophyton equinum CBS 127.97	F2PKV4
337	17001.879	Calmodulin	Trichophyton rubrum MR850	A0A022TTW9
338	17281.629	hypothetical protein H109_03367	Trichophyton interdigitale MR816	A0A059JA88
339	**17474.951**	**hypothetical protein TESG_03051**	**Trichophyton tonsurans CBS 112818**	**F2RWA2**
340	17574.482	40S ribosomal protein S15	Nannizzia gypsea CBS 118893	E4UY92
341	17592.703	Eukaryotic translation initiation factor 5A-2	Nannizzia gypsea CBS 118893	E4UQQ1
342	17602.514	40S ribosomal protein S15	Trichophyton rubrum CBS 288.86	A0A022VVB1
343	17604.492	40S ribosomal protein S15	Arthroderma otae CBS 113480	C5FX43
344	17663.789	Woronin body protein HexA, putative	Trichophyton verrucosum HKI 0517	D4DD77
345	17679.797	eukaryotic translation initiation factor 5A	Trichophyton rubrum D6	A0A059K392
346	17716.828	eukaryotic translation initiation factor 5A	Trichophyton interdigitale MR816	A0A059JCQ0
347	17928.84	hypothetical protein H109_04038	Trichophyton interdigitale MR816	A0A059J8S2
348	17939.832	hypothetical protein MGYG_02099	Nannizzia gypsea CBS 118893	E4UPP9
349	18026.865	conserved hypothetical protein	Arthroderma otae CBS 113480	C5FNM6
350	18045.832	hypothetical protein H109_06839	Trichophyton interdigitale MR816	A0A059J028
351	18075.826	60S ribosomal protein L21	Arthroderma otae CBS 113480	C5FZB3
352	18100.213	Nascent polypeptide-associated complex subunit beta	Trichophyton rubrum CBS 118892	F2SBS6
353	18105.836	60S ribosomal protein L21-A	Nannizzia gypsea CBS 118893	E4UW44
354	18342.914	Peptidyl-prolyl cis-trans isomerase, mitochondrial	Trichophyton interdigitale H6	A0A022UFH8
355	18439.168	hypothetical protein H107_08477	Trichophyton rubrum CBS 202.88	A0A023A328
356	18492.008	conserved hypothetical protein	Arthroderma otae CBS 113480	C5FFF0
357	18662.111	hypothetical protein H110_04932	Trichophyton rubrum MR1448	A0A022YLH9
358	18666.869	40S ribosomal protein S17-A	Trichophyton rubrum CBS 100081	A0A022UVW8
359	18668.213	hypothetical protein ARB_06761	Trichophyton benhamiae CBS 112371	D4ARL8
360	18695.355	RecName: Full=E3 ubiquitin ligase complex SCF subunit sconC	Microsporum canis	Q8TGW7
361	18718.15	hypothetical protein TEQG_07210	Trichophyton equinum CBS 127.97	F2Q2B4
362	18730.611	hypothetical protein TESG_03266	Trichophyton tonsurans CBS 112818	F2RWV1
363	18754.344	sulfur metabolite repression control protein C	Arthroderma otae CBS 113480	C5FHU9
364	18759.908	hypothetical protein ARB_05796	Trichophyton benhamiae CBS 112371	D4ANJ1
365	18810.299	E3 ubiquitin ligase complex SCF subunit sconC	Trichophyton rubrum MR1448	A0A022Z3F4

366	18853.348	hypothetical protein H109_00365	Trichophyton interdigitale MR816	A0A059JJL8
367	18877.605	hypothetical protein H103_06914	Trichophyton rubrum CBS 288.86	A0A022VTF3
368	18881.361	translationally controlled tumor protein	Trichophyton equinum CBS 127.97	F2PYM7
369	18906.658	Tropomyosin	Trichophyton equinum CBS 127.97	F2Q2U4
370	18911.346	hypothetical protein H105_00611	Trichophyton soudanense CBS 452.61	A0A022Y6V2
371	19015.793	hypothetical protein H101_06048	Trichophyton interdigitale H6	A0A022U8V1
372	19176.883	woronin body major protein	Trichophyton rubrum CBS 288.86	A0A022W205
373	19424.605	hypothetical protein TEQG_00992	Trichophyton equinum CBS 127.97	F2PJ84
374	19867.73	conserved hypothetical protein	Trichophyton benhamiae CBS 112371	D4B2I1
375	20367.268	hypothetical protein H109_04718	Trichophyton interdigitale MR816	A0A059J7B6
376	20380.986	hypothetical protein H100_03179	Trichophyton rubrum MR850	A0A022TS25
377	20399.24	hypothetical protein TEQG_00129	Trichophyton equinum CBS 127.97	F2PGQ7
378	20425.012	60S ribosomal protein L20	Trichophyton interdigitale MR816	A0A059JEI4
379	20531.004	30S ribosomal protein S3	Nannizzia gypsea CBS 118893	E4V353
380	20719.611	hypothetical protein H110_08115	Trichophyton rubrum MR1448	A0A022YBY5
381	20749.473	hypothetical protein H110_06661	Trichophyton rubrum MR1448	A0A022YGH1
382	20763.637	hypothetical protein H101_07261	Trichophyton interdigitale H6	A0A022U5G2
383	20871.463	hypothetical protein H102_05256	Trichophyton rubrum CBS 100081	A0A022V508
384	21068.971	hypothetical protein TRV_06972	Trichophyton verrucosum HKI 0517	D4DIG4
385	21112.191	40S ribosomal protein S15	Trichophyton tonsurans CBS 112818	F2RXI9
386	21121.727	NADH-quinone oxidoreductase	Trichophyton equinum CBS 127.97	F2PI29
387	21389.734	hypothetical protein TEQG_02780	Trichophyton equinum CBS 127.97	F2PPD0
388	21571.766	hypothetical protein H109_01768	Trichophyton interdigitale MR816	A0A059JG04
389	21625.814	cytochrome c subunit vb	Trichophyton tonsurans CBS 112818	F2S9E4
390	21734.984	NAD(P)H:quinone oxidoreductase, type IV	Trichophyton interdigitale MR816	A0A059JGJ7
391	21829.053	minor allergen Alt a 7	Trichophyton rubrum CBS 100081	A0A022VFI1
392	21852.023	flavodoxin	Trichophyton tonsurans CBS 112818	F2S3M0
393	23663.482	Adenine phosphoribosyltransferase	Trichophyton interdigitale MR816	A0A059JGG8
394	23693.492	Adenine phosphoribosyltransferase	Nannizzia gypsea CBS 118893	E4UZW4
395	23709.488	hypothetical protein ARB_04654	Trichophyton benhamiae CBS 112371	D4AK53
396	23709.488	Adenine phosphoribosyltransferase	Trichophyton rubrum CBS 118892	F2SU94

397	24064.018	nitroreductase	Trichophyton equinum CBS 127.97	F2PVJ3
398	24241.076	60S ribosomal protein L1	Arthroderma otae CBS 113480	C5FH50
399	24255.092	60S ribosomal protein L10A	Trichophyton interdigitale H6	A0A022U6Y0
400	25010.602	hypothetical protein H101_06572, partial	Trichophyton interdigitale H6	A0A022U872
401	25247.771	hypothetical protein H103_02885	Trichophyton rubrum CBS 288.86	A0A022W7V0
402	25250.762	hypothetical protein TEQG_06969	Trichophyton equinum CBS 127.97	F2Q1C0
403	25285.496	translation elongation factor 1 subunit Eef1-beta	Trichophyton equinum CBS 127.97	F2PMT1
404	25699.152	hypothetical protein TRV_02696	Trichophyton verrucosum HKI 0517	D4D6H1
405	25700.148	hypothetical protein TESG_07020	Trichophyton tonsurans CBS 112818	F2S7Z2
406	25713.168	hypothetical protein ARB_00424	Trichophyton benhamiae CBS 112371	D4AW59
407	25728.143	hypothetical protein H109_01174	Trichophyton interdigitale MR816	A0A059JHA5
408	25741.164	hypothetical protein H106_01464	Trichophyton rubrum CBS 735.88	A0A028JZX7
409	25769.195	hypothetical protein H103_01699	Trichophyton rubrum CBS 288.86	A0A022WBM3
410	26122.9	ThiJ/PfpI family protein	Trichophyton benhamiae CBS 112371	D4AMF4
411	26136.916	hypothetical protein TESG_07748	Trichophyton tonsurans CBS 112818	F2SA70
412	26200.879	ThiJ/PfpI family protein	Trichophyton verrucosum HKI 0517	D4D9S0
413	29855.912	hypothetical protein H104_02038	Trichophyton rubrum CBS 289.86	A0A022X6J9
