# Supplementary material for: Ultra-High-Resolution Mass Spectrometry for Identification of Closely Related Dermatophytes with Different Clinical Predilections
Source: J Clin Microbiol. 2018 Jun 25;56(7):e00102-18. doi: 10.1128/JCM.00102-18 (PMC6018324; doi:10.1128/JCM.00102-18)
Supplement: Supplemental material [file JCM.00102-18_zjm999095997s2.pdf]

Table ST2. Strain classification by algorithm A2.

rawfiles	prediction 1	prediction 2	prediction 3	prediction 4	CA
100084_Tr_1	100084_Tr	100084_Tr	100084_Tr	100084_Tr	1
100084_Tr_2	100084_Tr	100084_Tr	100084_Tr	100084_Tr	
100084_Tr_3	100084_Tr	100084_Tr	100084_Tr	100084_Tr	
100238_Tr_1	100238_Tr	100238_Tr	100238_Tr	100238_Tr	1
100238_Tr_2	100238_Tr	100238_Tr	100238_Tr	100238_Tr	
100238_Tr_3	100238_Tr	100238_Tr	100238_Tr	100238_Tr	
115314_Tr_1	115314_Tr	115314_Tr	115314_Tr	115314_Tr	1
115314_Tr_2	115314_Tr	115314_Tr	115314_Tr	115314_Tr	
115314_Tr_3	115314_Tr	115314_Tr	115314_Tr	115314_Tr	
118534_Tv_1	118534_Tv	118534_Tv	118534_Tv	118534_Tv	1
118534_Tv_2	118534_Tv	118534_Tv	118534_Tv	118534_Tv	
118534_Tv_3	118534_Tv	118534_Tv	118534_Tv	118534_Tv	
118892_Tr_1	118892_Tr	118892_Tr	118892_Tr	118892_Tr	1
118892_Tr_2	118892_Tr	118892_Tr	118892_Tr	118892_Tr	
118892_Tr_3	118892_Tr	118892_Tr	118892_Tr	118892_Tr	
119447_Tv_1	119447_Tv	119447_Tv	119447_Tv	119447_Tv	1
119447_Tv_2	119447_Tv	119447_Tv	119447_Tv	119447_Tv	
119447_Tv_3	119447_Tv	119447_Tv	119447_Tv	119447_Tv	
120316_Tv_1	120316_Tv	120316_Tv	120316_Tv	120316_Tv	1
120316_Tv_2	120316_Tv	120316_Tv	120316_Tv	120316_Tv	
120316_Tv_3	120316_Tv	120316_Tv	120316_Tv	120316_Tv	
120318_Tv_1	120318_Tv	120318_Tv	120318_Tv	120318_Tv	1
120318_Tv_2	120318_Tv	120318_Tv	120318_Tv	120318_Tv	
120318_Tv_3	120318_Tv	120318_Tv	120318_Tv	120318_Tv	
120320_Tv_1	120320_Tv	120320_Tv	120320_Tv	120320_Tv	1
120320_Tv_2	120320_Tv	120320_Tv	120320_Tv	120320_Tv	
120320_Tv_3	120320_Tv	120320_Tv	120320_Tv	120320_Tv	
201.88_Tv_1	201.88_Tv	201.88_Tv	201.88_Tv	201.88_Tv	1
201.88_Tv_2	201.88_Tv	201.88_Tv	201.88_Tv	201.88_Tv	
201.88_Tv_3	201.88_Tv	201.88_Tv	201.88_Tv	201.88_Tv	
202.88_Tr_1	202.88_Tr	202.88_Tr	202.88_Tr	202.88_Tr	1
202.88_Tr_2	202.88_Tr	202.88_Tr	202.88_Tr	202.88_Tr	
202.88_Tr_3	202.88_Tr	202.88_Tr	202.88_Tr	202.88_Tr	
288.86_Tr_1	288.86_Tr	288.86_Tr	288.86_Tr	288.86_Tr	1

288.86_Tr_2	288.86_Tr	288.86_Tr	288.86_Tr	288.86_Tr	
288.86_Tr_3	288.86_Tr	288.86_Tr	288.86_Tr	288.86_Tr	
452.61_Tv_1	452.61_Tv	452.61_Tv	452.61_Tv	452.61_Tv	1
452.61_Tv_2	452.61_Tv	452.61_Tv	452.61_Tv	452.61_Tv	
452.61_Tv_3	452.61_Tv	452.61_Tv	452.61_Tv	452.61_Tv	
318.31_Tt_1	318.31_Tt	318.31_Tt	318.31_Tt	318.31_Tt	1
318.31_Tt_2	318.31_Tt	318.31_Tt	318.31_Tt	318.31_Tt	
318.31_Tt_3	318.31_Tt	318.31_Tt	318.31_Tt	318.31_Tt	
285.30_Tt_1	285.30_Tt	285.30_Tt	285.30_Tt	285.30_Tt	1
285.30_Tt_2	285.30_Tt	285.30_Tt	285.30_Tt	285.30_Tt	
285.30_Tt_3	285.30_Tt	285.30_Tt	285.30_Tt	285.30_Tt	
100080_Te_1	100080_Te	100080_Te	100080_Te	100080_Te	1
100080_Te_2	100080_Te	100080_Te	100080_Te	100080_Te	
100080_Te_3	100080_Te	100080_Te	100080_Te	100080_Te	
856.71_Tt_1	856.71_Tt	856.71_Tt	856.71_Tt	856.71_Tt	1
856.71_Tt_2	856.71_Tt	856.71_Tt	856.71_Tt	856.71_Tt	
856.71_Tt_3	856.71_Tt	856.71_Tt	856.71_Tt	856.71_Tt	
109033_Tt_1	**634.82_Te**	109033_Tt	109033_Tt	109033_Tt	0.42
109033_Tt_2	109033_Tt	**634.82_Te**	**634.82_Te**	109033_Tt	
109033_Tt_3	**127.97_Tt**	**127.97_Tt**	**127.97_Tt**	**127.97_Tt**	
112186_Tt_1	112186_Tt	112186_Tt	112186_Tt	112186_Tt	0.92
112186_Tt_2	112186_Tt	112186_Tt	112186_Tt	112186_Tt	
112186_Tt_3	112186_Tt	**112198_Te**	112186_Tt	112186_Tt	
112188_Te_1	**270.66_Te**	**270.66_Te**	**270.66_Te**	**270.66_Te**	0.67
112188_Te_2	112188_Te	112188_Te	112188_Te	112188_Te	
112188_Te_3	112188_Te	112188_Te	112188_Te	112188_Te	
112193_Te_1	**112198_Te**	**112198_Te**	112193_Te	112193_Te	0.75
112193_Te_2	112193_Te	112193_Te	**270.66_Te**	112193_Te	
112193_Te_3	112193_Te	112193_Te	112193_Te	112193_Te	
112198_Te_1	112198_Te	112198_Te	112198_Te	112198_Te	0.67
112198_Te_2	**112193_Te**	**112193_Te**	**112193_Te**	**270.66_Te**	
112198_Te_3	112198_Te	112198_Te	112198_Te	112198_Te	
127.97_Tt_1	**109033_Tt**	127.97_Tt	**109033_Tt**	**109033_Tt**	0.42
127.97_Tt_2	**109033_Tt**	127.97_Tt	**109033_Tt**	**109033_Tt**	
127.97_Tt_3	127.97_Tt	**109033_Tt**	127.97_Tt	127.97_Tt	
270.66_Te_1	**112193_Te**	270.66_Te	**112193_Te**	**112198_Te**	0.42
270.66_Te_2	**112198_Te**	**112193_Te**	**112198_Te**	**112193_Te**	
270.66_Te_3	270.66_Te	270.66_Te	270.66_Te	270.66_Te	
634.82_Te_1	634.82_Te	634.82_Te	634.82_Te	634.82_Te	0.83
634.82_Te_2	634.82_Te	**109033_Tt**	**109033_Tt**	634.82_Te	
634.82_Te_3	634.82_Te	634.82_Te	634.82_Te	634.82_Te	
